# Impact of Surgical Delay on Two-Stage Breast Reconstruction During the COVID-19 Pandemic: A Retrospective Analysis

**DOI:** 10.3390/jcm14186684

**Published:** 2025-09-22

**Authors:** Ferruccio Paganini, Elisa Bascialla, Beatrice Corsini, Chiara Truini, Monica Arcaini, Lorenzo Fresta, Federico Lo Torto, Marco Marcasciano, Sara Matarazzo, Diego Ribuffo, Luigi Valdatta

**Affiliations:** 1 Department of Biotechnology and Life Sciences, Division of Plastic and Reconstructive Surgery, University of Insubria, Via Ravasi, 2, 21100 Varese, Italy; elisabascialla@gmail.com (E.B.); beatricecorsini97@gmail.com (B.C.); chiara.truini@unimi.it (C.T.); marcaini@studenti.uninsubria.it (M.A.); lfresta@studenti.uninsubria.it (L.F.); smsaramatarazzo@gmail.com (S.M.); luigi.valdatta@uninsubria.it (L.V.); 2 Department of Surgery “P. Valdoni”, Division of Plastic and Reconstructive Surgery, Policlinico Umberto I, Sapienza University of Rome, 00161 Rome, Italy; federico.lotorto@uniroma1.it (F.L.T.); diego.ribuffo@uniroma1.it (D.R.); 3 Department of Experimental and Clinical Medicine, Division of Plastic Surgery, Magna Graecia, University of Catanzaro, 88100 Catanzaro, Italy; m.marcasciano@unicz.it

**Keywords:** breast reconstruction, tissue expander, COVID-19 pandemic, surgical delay, capsular contracture, patient-reported outcomes

## Abstract

**Background:** The COVID-19 pandemic caused unprecedented delays in elective surgery, including breast reconstruction, prolonging expander retention beyond recommended timelines. **Methods:** We retrospectively compared patients who underwent two-stage expander-to-implant reconstruction before the pandemic (2011–2020) and during the pandemic (2020–2022). Clinical outcomes and patient-reported experiences were analyzed, and multivariate regression was used to adjust for confounders. **Results:** Expander retention was significantly longer in the pandemic cohort (481 vs. 280 days). Capsular contracture around the expander was markedly increased, with pandemic group assignment and prolonged expander retention emerging as independent predictors in multivariate analysis, while overall complication rates were unaffected. Patient-reported outcomes showed more functional limitations but paradoxically higher satisfaction with the definitive implant. **Conclusions:** Surgical delay selectively increased the risk of expander contracture without raising overall morbidity. Patient-reported findings highlight the dual impact of delay, with both greater burden and a potential “relief effect”.

## 1. Introduction

The COVID-19 pandemic forced surgical units worldwide to delay non-urgent procedures, including reconstructive surgery [1,2,3]. Breast reconstruction, though not lifesaving, plays a critical role in oncological recovery, body image, and quality of life. The unprecedented disruption of surgical schedules created a unique context to evaluate how delayed timelines affect outcomes in expander-based reconstruction.

Breast reconstruction has long been debated in the literature [4,5,6,7,8,9,10], particularly regarding timing (immediate vs. delayed) and reconstructive methods (alloplastic, autologous, or hybrid). In high-income countries, reconstruction following mastectomy is considered an integral part of breast cancer treatment, not only for aesthetic restoration but also as an element of personalized care within precision medicine [11].

During the pandemic (2020–2022) [12,13], resource allocation and hospital reorganization led to significant delays in second-stage procedures. Expander-based reconstruction, already widely used, became a pragmatic option to provide at least temporary restoration [14,15], preventing prolonged amastia and its psychological consequences. However, placing expanders without the possibility of timely exchange inevitably prolonged retention, raising concerns about device-related complications, surgical outcomes, and patient discomfort.

This study leverages the pandemic as an unprecedented “natural experiment” to investigate the effects of delayed expander-to-implant exchange. We aimed to (i) quantify the delay imposed by the pandemic, (ii) evaluate early and late surgical complications, and (iii) explore patient-reported outcomes to assess the functional and psychosocial impact of prolonged expander retention.

## 2. Patients and Methods

### 2.1. Study Design

This was a retrospective, single-center observational study conducted at the Department of Plastic and Reconstructive Surgery of the Circolo Hospital and Macchi Foundation in Varese, Italy. The study population consisted of all patients who underwent two-stage alloplastic breast reconstruction with a tissue expander followed by exchange to a permanent implant between December 2011 and December 2022. Data collection and analysis were performed between June and September 2024.

To ensure adequate follow-up for the assessment of late complications, only patients with at least 18 months of follow-up after the second-stage procedure were included. Patients were excluded if they

- underwent autologous reconstruction at the second stage,
- received direct-to-implant reconstruction or alternative reconstructive approaches,
- required early expander-to-implant exchange for oncological reasons (<6 months),
- had implant removal without reconstruction, or
- were lost to follow-up.

The study design was chosen to compare two naturally occurring cohorts:

1. Case group (pandemic cohort): patients scheduled for the second-stage reconstruction between February 2020 (declaration of the COVID-19 emergency in Italy) and December 2022.
2. Control group (pre-pandemic cohort): patients who completed the second-stage reconstruction between December 2011 and February 2020, before the pandemic.

This setting provided a unique opportunity to analyze the effects of surgical delay imposed by a global health crisis. Our center was not designated as a COVID-free facility; consequently, surgical resources were frequently diverted to pandemic care, leading to unavoidable delays in expander-to-implant exchange.

The study was conducted in accordance with the STROBE guidelines for observational research [16]. Ethical approval was obtained from the institutional review board, and informed consent was acquired from all participants.

### 2.2. Variables Analysed

A wide range of variables were collected to compare the pre-pandemic and pandemic cohorts and to evaluate the impact of surgical delay. Data were retrieved from electronic medical records and organized into the following categories:

- Demographic variables: age, body mass index (BMI), menopausal status, smoking habits, comorbidities (diabetes, hypothyroidism, hypercholesterolemia, arterial hypertension), and lifestyle factors.
- Oncological variables: tumor histology and stage, BRCA mutation status, type of mastectomy (modified radical, skin-sparing, nipple-sparing), axillary management (sentinel node biopsy, axillary dissection), and the need for neoadjuvant or adjuvant therapies (chemotherapy, radiotherapy).
- Perioperative variables (first stage): pre- and postoperative hemoglobin and hematocrit, use of acellular dermal matrix (ADM), number and type of drains, length of hospital stay, time to drain removal, expansion protocol (time to first expansion, expansion duration, final expansion volume), and type of expander used.
- Reconstructive variables (second stage): plane of implant placement, type and size of definitive implant, use of contralateral symmetrization procedures, and length of hospital stay at exchange.
- Outcomes:
  ○ Early complications (within 30 postoperative days): hematoma, seroma, infection, wound dehiscence, need for reoperation, implant loss.
  ○ Late complications (beyond 30 days until expander replacement): rupture, capsular contracture, infection, seroma, wound dehiscence, implant exposure, reoperation.
  ○ Capsular contracture after definitive implant exchange (minimum 18 months follow-up).

This structured dataset allowed for comparison of demographic and clinical factors between groups, evaluation of early and late complications, and identification of predictors of adverse outcomes.

### 2.3. Surgical Protocols

Surgical indications for expander-based reconstruction were established on the basis of oncological treatment plans, breast anatomy, mastectomy flap viability, patient comorbidities, and overall compliance.

In the first stage, a tissue expander was inserted into a subpectoral pocket, with an acellular dermal matrix (ADM) used in selected cases to cover the lower pole. A drain was positioned partly in the subpectoral pocket and partly in the subcutaneous layer; in patients undergoing complete axillary dissection, a second drain was placed in the axillary cavity.

In the second stage, the expander was removed and the periprosthetic capsule remodelled with capsulotomies or partial capsulectomy when required. A permanent implant was then inserted. Symmetry procedures on the contralateral breast (reduction, augmentation, or mastopexy) were performed when indicated and requested by the patient. A single drain was placed during exchange procedures.

### 2.4. Perioperative Management and Follow-Up

All patients were treated according to standardized institutional protocols, which remained unchanged during the entire study period.

- Antibiotic prophylaxis: intravenous cefazolin (or clindamycin in allergic patients) administered perioperatively, followed by extended prophylaxis with oral amoxicillin–clavulanic acid (or ciprofloxacin in allergic patients) until drains were removed.
- Analgesia: scheduled paracetamol 1000 mg three times daily; rescue analgesics included ketorolac 30 mg IV, ibuprofen 600 mg, or morphine 5 mg SC. Antiemetics were administered as needed, and proton pump inhibitors (pantoprazole or lansoprazole) were routinely prescribed.
- Anticoagulation: enoxaparin sodium 4000 IU daily for six days postoperatively.

The first dressing change after discharge was performed 3–4 days postoperatively, followed by twice-weekly dressing changes until drain removal. Scheduled follow-up visits took place 3 and 6 months after the expansion phase, and then every 6 months for at least 18 months after definitive implant placement.

### 2.5. Discomfort Assessment for Delays

To complement clinical outcomes, we evaluated patient-reported experiences during the expander retention period and after definitive implant placement. For this purpose, a nine-item questionnaire was specifically developed for the study (Supplementary Figure S1). The questionnaire was originally drafted in Italian and subsequently adapted into English for analysis.

The tool investigated three domains:

- Physical discomfort (e.g., pain, restrictions in daily activities, sleep quality),
- Psychosocial impact (e.g., body image, limitations in social or occupational activities),
- Satisfaction with final reconstruction (perceived adaptation to the implant, willingness to undergo the procedure again).

Each item was scored on a categorical scale, and responses were collected during scheduled follow-up visits after completion of the second stage of reconstruction.

The questionnaire was not a validated instrument such as the BREAST-Q, since standardized tools require preoperative baseline data and do not specifically capture retrospective perceptions of surgical delay. Instead, it was designed to address the unique situation created by the COVID-19 pandemic, aiming to explore patient perspectives that would otherwise remain undocumented.

### 2.6. Statistical Analysis

All data were entered into a dedicated database and analyzed using standard statistical software SPSS v30. Descriptive statistics were used to summarize demographic and clinical variables. Continuous variables were expressed as means with standard deviation (SD) and compared using Student’s *t*-test. Categorical variables were expressed as frequencies and percentages and compared using the chi-square test or Fisher’s exact test, as appropriate. Odds ratios (OR) with 95% confidence intervals (CI) were calculated to quantify the risk of specific complications.

Postoperative complications were analyzed by timing (early, within 30 days, vs. late, beyond 30 days until expander replacement) and by reconstructive stage (expander vs. definitive implant). Correlation analyses (Spearman’s rho) were performed to evaluate associations between expander maintenance time and questionnaire scores.

To account for potential confounders, a multivariate logistic regression model was built with capsular contracture as the primary outcome. Predictors included group assignment (pandemic vs. pre-pandemic), expander duration, age, smoking status, comorbidities, radiotherapy during expansion, and follow-up length. A secondary logistic regression was performed using “any complication” as the outcome. Results were reported as adjusted ORs with 95% CI.

A *p*-value < 0.05 was considered statistically significant for all analyses.

## 3. Results

Between December 2011 and April 2022, a total of 366 expander-based breast reconstructions were performed at our institution. Of these, 147 patients underwent the second-stage procedure during the COVID-19 pandemic (Group A, cases), and 219 patients completed reconstruction in the pre-pandemic period (Group B, controls).

In the case group, 142 patients received expander-to-implant exchange between September 2020 and April 2022, reflecting the delay imposed by the suspension of elective surgery in the first pandemic months (February–August 2020). In the control group, 215 patients completed reconstruction between 2011 and February 2020.

This division allowed for comparison of two naturally occurring cohorts of considerable size, providing one of the largest single-center analyses to date on the impact of surgical delay in two-stage breast reconstruction and strengthening the generalizability of our findings.

### 3.1. Demographic Analysis (Supplementary Table S1)

The mean age was 49 years (range 30–76) in the pandemic group compared with 53 years (range 25–75) in the pre-pandemic group. This difference was statistically significant (*t* = −3.01, *p* = 0.003), indicating that patients undergoing reconstruction during the pandemic were on average younger. This imbalance represents a potential confounding factor for subsequent analyses and was therefore included in the multivariate regression models.

Apart from age, no significant demographic or clinical differences were observed between groups. Rates of comorbidities such as diabetes, hypothyroidism, hypercholesterolemia, and arterial hypertension were similar, as were pre- and postoperative hemoglobin and hematocrit values (Supplementary Table S2). Tumor histology and staging, BRCA mutation status, and oncological treatments (chemotherapy, radiotherapy) did not differ significantly between cohorts (Supplementary Table S3, Table 1).

Reconstructive characteristics, including type of expander, duration of expansion, and final expansion volume, were also comparable across groups (Table 2). Similarly, perioperative outcomes such as hospital stay and time to drain removal did not differ significantly.

### 3.2. Treatment Delays (Table 2)

The interval between the first and second stages of reconstruction was markedly longer in the pandemic cohort compared with the pre-pandemic group. Patients in the case group experienced an average waiting time of 481 days (SD 231), whereas controls underwent exchange after a mean of 280 days (SD 137). This difference was highly significant (t = 10.35, *p* < 0.00001).

These data confirm the profound impact of the COVID-19 pandemic on reconstructive scheduling. The increase of more than 200 days in the average waiting period highlights the systemic delays imposed by resource reallocation and restrictions on elective procedures, which created the unique conditions for this natural experiment.

### 3.3. Complication Analysis (Table 3, Supplementary Figures S2 and S3)

A total of 122 complications were observed across the entire cohort of 366 patients. Complications occurred in 43 patients in each group, but their distribution differed significantly between cohorts. The overall complication rate was higher in the pandemic group (29%) compared with the pre-pandemic group (18%), with chi-square analysis confirming statistical significance (χ^2^ = 4.52, *p* = 0.033).

**Table 3:** Complications analysis.

	Group 1 (Cases)	Group 2 (Controls)	*p* Value *
Total complications	29% (43)	18% (43)	0.0333
Early complications (<30 days)			
Hematoma	4% (6)	2% (5)	0.3592
RTOR	0% (0)	0.5% (1)	
Seroma	10% (14)	5% (12)	0.1499
RTOR	0% (0)	0% (0)	
Infection	5% (7)	3% (6)	0.3892
RTOR	2% (3)	1% (2)	
Wound dehiscence	8% (12)	10% (21)	0.0715
RTOR	2% (3)	1% (3)	
Implant explant	1% (2)	2% (5)	0.7068
Late complications (>30 days)			
Implant rupture	2% (3)	0.5% (1)	0.3055
Contracture	7% (10)	1% (3)	0.0077 **
Hematoma	0% (0)	0% (0)	1
RTOR	0% (0)	0% (0)	
Seroma	1% (1)	0.5% (1)	1
RTOR	0% (0)	0% (0)	
Infection	0% (0)	1% (2)	0.5196
RTOR	0% (0)	0.5% (1)	
Wound dehiscence	3% (4)	0.5% (1)	0.0839
RTOR	1% (2)	0% (0)	
Implant exposure	0% (0)	0% (0)	1
RTOR	0% (0)	0% (0)	
Implant explant	2% (3)	1% (3)	0.6856

Legend: RTOR—Return To Operating Room; *—at Fisher exact test; ** statistically significant.

When stratified by timing, early complications (within 30 days) did not differ significantly between groups (Supplementary Figure S2). By contrast, late complications (beyond 30 days until expander replacement) were significantly more frequent in the pandemic cohort (Supplementary Figure S3), mainly driven by capsular contracture around the expander (χ^2^ = 7.71, *p* = 0.005).

**Figure 1:**
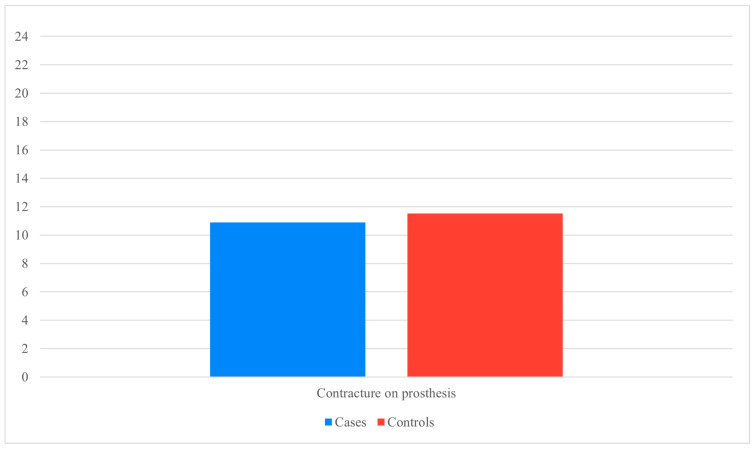
Contracture on definitive prosthesis (Data presented as percentages. Y-axis scaled to data range for readability).

Capsular contracture emerged as the complication most strongly associated with surgical delay. The odds ratio for developing contracture during the expander phase was 5.32 in the pandemic group, compared with 1.2 after definitive implant exchange. Notably, once the expander was replaced with a permanent implant, the difference in contracture rates was no longer statistically significant (χ^2^ = 0.24, *p* = 0.621) (Figure 1).

In addition, the rate of expander rupture was higher in the pandemic group, although this difference did not reach statistical significance. Other late complications, including infection, seroma, and wound dehiscence, occurred at similar frequencies across groups.

#### Multivariate Analysis (Table 4)

Multivariate logistic regression was performed to account for potential confounding factors, including age, smoking status, comorbidities, radiotherapy during expansion, and follow-up duration (See Table 4 for the full multivariate logistic regression model).

**Table 4:** Multivariate logistic regression analysis for predictors of complication.

Outcome	Predictor	OR	95% CI	*p*-Value
Capsular contracture (Model A)	Pandemic group (vs. pre-pandemic)	33.4	0.68–63.4	0.015
	Age	1.01	-	0.18 (ns)
	Smoking	1.08	-	0.81 (ns)
	Comorbidities	0.99	-	≈1.0 (ns)
	Radiotherapy during expansion	-	-	ns
	Follow-up (months)	1.05	-	0.078 (trend)
Capsular contracture (Model B)	Expander duration (per day)	1.006	1.002–1.009	0.002
	(other covariates)	-	-	ns
Any complication (Model C)	Pandemic group (vs. pre-pandemic)	1.54	0.82–4.04	0.167
	Age	1.02	-	0.12 (ns)
	Comorbidities	0.58	-	0.12 (ns)
	Follow-up (months)	1.014	1.000–1.027	0.047
	Smoking, Radiotherapy	-	-	ns
Any complication (Model D)	Expander duration (per day)	1.0003	0.999–1.002	0.66
	Follow-up (months)	1.012	-	0.076 (trend)
	(other covariates)	-	-	ns

ns = not significant; OR = Odds Ratio; CI = Confidence Interval. Models A and C included pandemic group as main predictor; Models B and D included expander retention time as a continuous variable instead, to avoid collinearity.

When capsular contracture around the expander was set as the primary outcome, belonging to the pandemic group remained an independent predictor (OR 33.4, 95% CI 0.68–63.4, *p* = 0.015). Although the odds ratio was very large—reflecting both the strong association between surgical delay and contracture and the limited number of events—this result reinforces the clinical relevance of delay as a risk factor, rather than representing a statistical artifact.

When group assignment was replaced by expander retention time as a continuous variable, longer duration was likewise an independent predictor of capsular contracture (OR 1.006 per day, 95% CI 1.002–1.009, *p* = 0.002). This finding reinforces the conclusion that surgical delay itself, rather than demographic or oncological variables, was the main determinant of contracture risk.

By contrast, when “any complication” was considered as the outcome, neither group assignment (OR 1.54, *p* = 0.167) nor expander duration (OR 1.0003 per day, *p* = 0.66) showed significant associations. The only variable independently associated with complication reporting was follow-up length (OR 1.014 per month, 95% CI 1.000–1.027, *p* = 0.047), likely reflecting a surveillance bias, whereby longer observation increases the chance of identifying at least one complication.

Taken together, these analyses indicate that prolonged expander retention specifically increases the risk of capsular contracture, while not contributing to a broader rise in postoperative morbidity.

Clinically, this means that surgical delay was the predominant driver of expander contracture, while other patient- and treatment-related factors had only a marginal role.

### 3.4. Reoperation Rates

Reoperations for complications were required in both groups at comparable rates. Overall, 17% of patients with early complications and 10% of those with late complications underwent surgical revision, with no significant difference between the pandemic and pre-pandemic cohorts. These findings indicate that, despite the higher incidence of expander contracture in delayed patients, the overall burden of reoperation was not increased.

A notable clinical vignette further illustrates the patient perspective. One patient with a history of fibromyalgia experienced persistent pain during the prolonged expander phase and, faced with repeated delays in proceeding to the second stage, ultimately chose explantation without further reconstruction. This case underscores how subjective experience and comorbid conditions can influence surgical outcomes beyond standard complication metrics.

Reoperation rates observed in this series are consistent with those reported in the literature for expander–implant reconstruction, supporting the generalizability of our findings.

To further qualify the severity of complications, events were classified according to the Clavien–Dindo system [17,18].

### 3.5. Classification of Complications Using the Clavien-Dindo System [17,18]

Complications were graded according to the Clavien–Dindo classification to better capture their clinical severity.

Among early complications, the majority were minor (Grade 1–2), including wound healing problems, seroma, or infections managed conservatively. Grade 3b complications requiring surgical intervention were observed in 8 patients in the pandemic group and 11 in the pre-pandemic group.

Late complications followed a similar distribution, with most cases classified as Grade 1–2 and only a minority requiring reoperation (Grade 3b: 5 cases in the pandemic group vs. 4 in the control group).

No statistically significant differences were observed between groups in the distribution of complication grades (Fisher’s exact test, *p* > 0.05).

These findings confirm that, although surgical delay increased the incidence of capsular contracture, it did not shift the overall severity profile of complications. Most adverse events remained low-grade and manageable with conservative treatment, and the rates of severe complications requiring surgical revision were comparable between cohorts.

### 3.6. Patient Questionnaire (Supplementary Figure S1)

**Figure 2:**
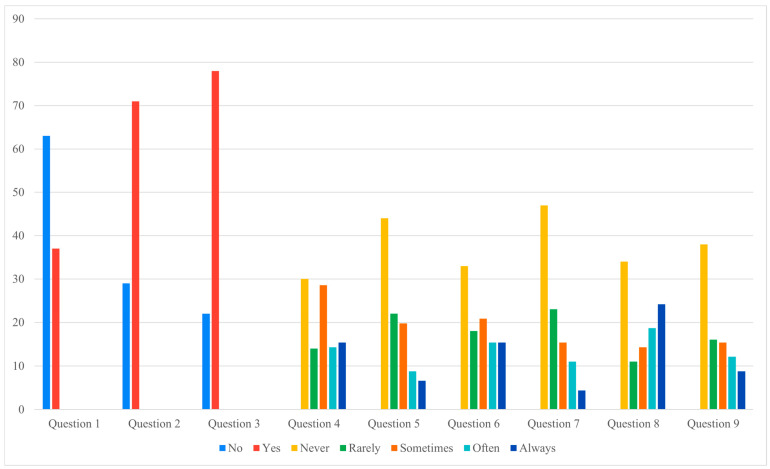
Cases questionnaire answers (Data presented as percentages. Y-axis scaled to data range for readability).

**Figure 3:**
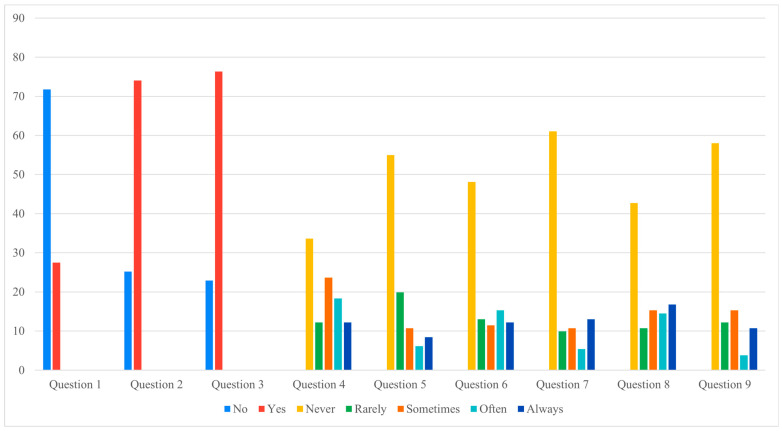
Controls questionnaire answers (Data presented as percentages. Y-axis scaled to data range for readability).

Analysis of the nine-item questionnaire revealed that most responses did not differ significantly between groups (Figure 2 and Figure 3). However, a notable difference was observed for Question 7, which investigated physical or occupational limitations related to the definitive implant (e.g., need to change sports or job activities). Patients in the pandemic group reported significantly greater limitations, with a chi-square value of 15.23 (*p* = 0.004).

Correlation analyses further highlighted the impact of prolonged expander retention on patient-reported outcomes. In the pandemic cohort, longer expander duration was directly associated with higher discomfort scores (Question 6, rs = 0.2, *p* = 0.03) and with negative perceptions of reconstruction (Question 2, rs = 0.3, *p* = 0.002; Question 3, rs = 0.2, *p* = 0.05). Conversely, inverse correlations were observed for sleep quality (Question 5, rs = −0.4, *p* = 0.0005) and return to normal habits (Question 9, rs = −0.2, *p* = 0.02). No significant correlations were found in the control group.

Taken together, these results indicate that prolonged expander maintenance had a measurable negative impact on patients’ daily lives, increasing functional limitations and discomfort. At the same time, the paradoxical finding of higher satisfaction with the definitive implant among delayed patients suggests a more complex psychological response, which is further explored in the Discussion.

Figure 2 and Figure 3 illustrate these findings graphically, showing that, apart from the significant difference in Question 7, most questionnaire responses were comparable between the two cohorts. This visual consistency supports the interpretation that prolonged expander retention influenced specific functional domains rather than broadly altering patient perceptions.

## 4. Discussion

This study aimed to investigate potential complications following mastectomy and breast reconstruction, particularly associated with the prolonged retention of tissue expanders beyond six months—the timeframe generally recommended in the scientific literature [19]. Literature indicates that the optimal duration for expander retention is approximately six months, although no absolute limit is defined [20,21,22,23]. This period is considered sufficient to complete tissue expansion and to minimize complications such as capsular contracture, thereby optimizing both aesthetic and functional outcomes in breast reconstruction [20].

Other studies have suggested that extending expander retention beyond six months may increase the risk of complications, including capsular contracture and infection [24], based on clinical experience and observational reports documenting higher complication rates in patients with prolonged expansion [25,26]. At the same time, recent work has highlighted the potential benefits of one-stage reconstruction (e.g., prepectoral or dual-plane approaches) when clinically feasible, as these strategies avoid a second operation and may reduce costs and recovery time.

Against this background, our study leverages the COVID-19 pandemic as a “natural experiment” that forced systematic delays in surgical scheduling. With one of the largest single-center cohorts analyzed to date, we were able to demonstrate that prolonged expander retention selectively increased the risk of capsular contracture, while overall complication rates remained unaffected. This selective effect adds new evidence to the literature, showing that surgical delay primarily impacts contracture rather than general morbidity.

### 4.1. Significance of Data

Expander-based two-stage reconstruction has long been associated with timing-related outcomes. Literature generally recommends limiting expander retention to about six months [19,20,21,22,23]. Extending this period has been linked to increased rates of contracture and infection [24,25,26]. Our results confirm this risk: patients whose exchange was delayed beyond six months showed a significantly higher incidence of capsular contracture, consistent with previous reports [20,21,22,23,24,25,26,27,28]. Importantly, multivariate regression confirmed that both pandemic group assignment and expander duration as a continuous variable independently predicted contracture, underscoring surgical delay itself as the main determinant.

By contrast, no significant increase in overall complications was observed. Early postoperative outcomes remained stable, likely reflecting the consistent application of perioperative protocols (antibiotic prophylaxis, drain management, analgesia, anticoagulation) that were maintained throughout the pandemic. This suggests that systemic disruption affected scheduling rather than the quality of perioperative care. Device integrity remains a concern: manufacturers warn that shell fatigue and folding may occur when expanders are retained beyond recommended times [29,30,31]. While rupture rates were not statistically different, the higher trend in the delayed group supports this biological plausibility and reinforces the need for careful monitoring.

This selective effect of delay—impacting contracture but not overall complication rates—emerges as the novel contribution of our study, distinguishing it from previous literature where global morbidity was often emphasized.

### 4.2. Patient Questionnaire Analysis (Supplementary Figure S1)

Beyond complications, surgical delay profoundly shaped patient experience. Our custom questionnaire—developed to address perceptions of delay, given that validated instruments such as the BREAST-Q require preoperative data and do not specifically capture retrospective experiences—revealed both detrimental and paradoxical effects.

Negatively, patients in the delayed group reported greater physical and occupational limitations (Question 7), in line with prior evidence that prolonged expander use may cause chronic pain, functional impairment, and reduced upper-limb mobility [27,32,33,34]. This underlines the functional burden imposed by extended retention, which is not reflected by complication rates alone.

At the same time, paradoxical responses emerged. Despite reporting more discomfort, delayed patients expressed greater satisfaction with the definitive implant. Two explanations are possible. First, longer retention may promote genuine adaptation to the prosthetic device, facilitating eventual acceptance. Second, and perhaps more plausibly, this pattern may reflect a “relief effect”: after enduring prolonged discomfort and uncertainty, patients perceive the final reconstruction more positively, independent of objective outcomes. Both mechanisms are relevant and highlight the complexity of patient perception under disrupted timelines.

Correlation analyses supported this duality. Prolonged retention correlated with higher discomfort (Question 6), but inversely with sleep quality (Question 5) and return to normal habits (Question 9). These findings suggest that delay negatively affects daily well-being during expansion, yet the eventual transition to a definitive implant is perceived as particularly rewarding. Such nuances should be considered in patient counseling, especially when further delays are anticipated.

### 4.3. Comparison with Literature

Our results strengthen existing evidence that expander retention beyond six months increases contracture risk [20,21,22,23,24,25,26,28,35]. The multivariate approach adds weight by confirming surgical delay itself, rather than patient- or tumor-related factors, as the main driver.

In contrast, infection and seroma rates were not increased, diverging from some reports linking prolonged expansion to higher morbidity [24,25]. Uniform institutional protocols may explain this difference. Likewise, overall complication rates remained unchanged, consistent with other studies suggesting that two-stage reconstruction can be safe even in challenging contexts [21,36,37,38,39].

Concerns about device integrity have been raised in both clinical and experimental studies, with reports of expander rupture and shell weakness after long-term use [29,30,31,40]. Our data showed no significant difference, but the observed trend toward more ruptures in delayed cases aligns with these warnings.

Finally, radiotherapy—commonly identified as a major risk factor for adverse prosthetic outcomes [41,42,43]—was not associated with increased contracture in our cohort. This discrepancy may reflect the relatively small number of irradiated patients, limiting statistical power. Thus, while our findings suggest delay is the dominant driver of contracture, the role of radiotherapy should not be underestimated in broader populations.

### 4.4. Decision-Making Impact

The COVID-19 pandemic acted as a “stress test” for reconstructive surgery. Our results show that prolonged expander retention selectively increased contracture risk without amplifying overall morbidity. Clinically, this means that when delays are unavoidable, expander-based reconstruction remains a viable and safe temporizing option—provided that patients are informed of the higher likelihood of contracture and monitored accordingly.

These data also contribute to the ongoing debate about reconstructive timing. One-stage direct-to-implant or autologous procedures may circumvent the risks of prolonged expansion, but two-stage reconstruction retains value in resource-limited or emergency contexts, where shorter operative times and scheduling flexibility are critical [14,15,21,36,37,38,39].

The paradoxical increase in satisfaction among delayed patients underscores the importance of integrating PROs into decision-making. Surgeons must not only weigh risks and benefits but also address the psychological impact of delay, acknowledging that patient adaptation and relief strongly influence satisfaction. Transparent counseling can help align expectations and optimize outcomes, even under non-standard conditions.

### 4.5. Study Limitations

This study has limitations. Its retrospective design precludes causal inference and may leave residual confounding despite multivariate adjustment. The significant age difference between groups represents another limitation, although age was included in regression models.

Patient-reported outcomes were assessed using a custom, non-validated tool. While this lacks the psychometric rigor of validated instruments such as the BREAST-Q, it was specifically designed to capture the retrospective perception of delay, which standardized questionnaires cannot address.

We did not analyze granular data on perioperative medications (NSAIDs, anticoagulants, analgesics), which may influence wound healing and complications. However, consistent institutional protocols reduce the likelihood of systematic bias. Follow-up duration was slightly longer in controls, which may have led to greater detection of late complications in that group.

Despite these limitations, this study provides one of the first real-world analyses of pandemic-related reconstructive delays, integrating both clinical outcomes and patient-reported measures. These insights can inform both clinical counseling and future guidelines for breast reconstruction in extraordinary healthcare circumstances.

## 5. Conclusions

This study demonstrates that surgical delay during the COVID-19 pandemic, with expander retention beyond the recommended six months, selectively increased the risk of capsular contracture while not significantly affecting the overall complication burden. These findings suggest that delay acts as a specific rather than generalized risk factor.

Patient-reported outcomes highlighted the dual nature of this experience: greater physical limitations during expander maintenance but paradoxically higher satisfaction with the final implant, likely reflecting both improved adaptation and a “relief effect.” This underlines the importance of integrating objective outcomes with the patient perspective.

From a clinical standpoint, prolonged expander retention can be considered a safe temporizing strategy in extraordinary circumstances, provided that patients are carefully counseled about the higher likelihood of contracture and closely monitored throughout follow-up. Anticipating this risk and addressing expectations transparently may help preserve satisfaction even when standard timelines cannot be respected.

These results offer preliminary evidence to inform future guidelines on reconstructive timing in crisis situations. Prospective, multicenter studies incorporating validated patient-reported outcome measures are warranted to confirm these findings and refine recommendations for both routine and emergency settings.

## Figures and Tables

**Table 1:** Cancer treatment analysis.

	Group 1 (Cases)	Group 2 (Controls)
Pre-mastectomy oncological treatment		
Neo-adiuvant chemotherapy	16% (24)	19% (41)
Radiotherapy	3% (5)	6% (13)
Mastectomy type		
Modified radical	62% (91)	64% (140)
Skin-sparing	5% (7)	4% (8)
Nipple-sparing	33% (48)	32% (69)
Nodes treatment		
Sentinel node biopsy	78% (115)	67% (147)
Axillary dissection	21% (31)	32% (70)
Post-mastectomy oncological treatment		
Adiuvant chemotherapy	24% (35)	32% (71)
Radiotherapy on breast expander	3% (5)	2% (4)

**Table 2:** Reconstruction analysis.

	Group 1 (Cases)	Group 2 (Controls)	*p* Value
Plane of breast expander positioning			
Retro-pectoral	98% (144)	95% (208)	N.A.
Retro-pectoral with ADM	1% (2)	4% (9)	N.A.
Breast expander volume			
250 cc	1% (1)	1% (2)	N.A.
275 cc	1% (2)	3% (7)	N.A.
350 cc	14% (21)	18% (39)	N.A.
450 cc	33% (48)	29% (63)	N.A.
550 cc	39% (57)	29% (64)	N.A.
650 cc	12% (17)	19% (42)	N.A.
Breast expander type			
LH	3% (4)	3% (7)	N.A.
MH	97% (142)	96% (210)	N.A.
TH	0% (0)	0.5% (1)	N.A.
Mean hospital stay without complications (days)	5	5	0.2502
Mean total number of drains	1	1	1
Mean time active drains keeping (days)	14	21	0.0146 *
Mean time from surgery to first expansion (days)	22	25	0.0802
Mean expansion time without complications (days)	98	66	0.0012 *
Final expansion volume			
Under size	37% (54)	33% (72)	N.A.
Right size	38% (56)	38% (84)	N.A.
Over size	23% (34)	24% (53)	N.A.
Mean time to second stage procedure (days)	481	280	<0.00001 *
Mean follow-up after second stage procedure (months)	28	33	0.0129

Legend: ADM—Acellular Dermal Matrix; N.A.—not applicable; *—statistical significant at *t*-test.

## Data Availability

No data collection forms, extracted data, analytic code or other materials used in this review are publicly available, but are available from the corresponding author upon reasonable request.

## Supplementary Materials

The following supporting information can be downloaded at: https://www.mdpi.com/article/10.3390/jcm14186684/s1, Figure S1: Questions module (English version adapted form original version in Italian language); Figure S2: Early complications; Figure S3: Late complications; Table S1: Demographic analysis; Table S2: Peri-operative hemogram analysis; Table S3: Oncological analysis.
