# Supplementary material for: Impact of Surgical Delay on Two-Stage Breast Reconstruction During the COVID-19 Pandemic: A Retrospective Analysis
Source: J Clin Med. 2025 Sep 22;14(18):6684. doi: 10.3390/jcm14186684 (PMC12470722; doi:10.3390/jcm14186684)
Supplement: Supplementary file 1 [file jcm-14-06684-s001.zip › jcm-3868916-supplementary.pdf]

Patient____________________Date of Birth__________Phone number____________________________

Question	Answer				
1. During the stay of the expander, has the discomfort or pain remained the same or worsened?	1 NO	2 YES			
2. Did the sensation of discomfort or pain you had before resolve with the replacement of the expander with the prosthesis?	1 NO	2 YES			
3. Given your experience, would you repeat this type of reconstruction again?	1 NO	2 YES			
4. Did you struggle to sleep with the expander?	1 NEVER	2 RARELY	3 SOMETIMES	4 USUALLY	5 ALWAYS
5. Do you have trouble sleeping with the prosthesis?	1 NEVER	2 RARELY	3 SOMETIMES	4 USUALLY	5 ALWAYS
6. With the expander, did you have limitations in your physical or work activity (such as, for example, changing sports or jobs)?	1 NEVER	2 RARELY	3 SOMETIMES	4 USUALLY	5 ALWAYS
7. With the prosthesis, do you have limitations in your physical activity or work (such as, for example, changing sports or jobs)?	1 NEVER	2 RARELY	3 SOMETIMES	4 USUALLY	5 ALWAYS
8. With the expander, did you have to change some of your lifestyle habits (such as the way you dress or wearing a swimsuit)?	1 NEVER	2 RARELY	3 SOMETIMES	4 USUALLY	5 ALWAYS
9. With the prosthesis, have you changed some of your lifestyle habits (such as the way you dress or wearing a swimsuit)?	1 NEVER	2 RARELY	3 SOMETIMES	4 USUALLY	5 ALWAYS

Figure S1. Questions module (English version adapted form original version in Italian language).

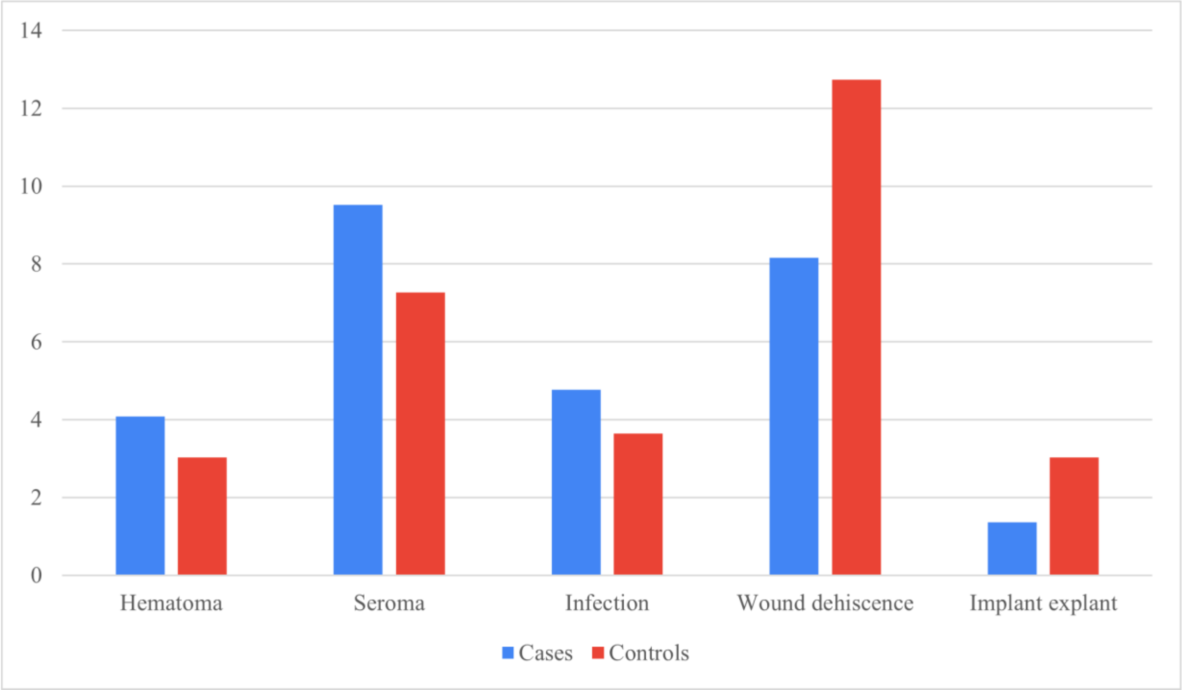

Figure S2. Early complications (Data presented as percentages. Y-axis scaled to data range for readability).

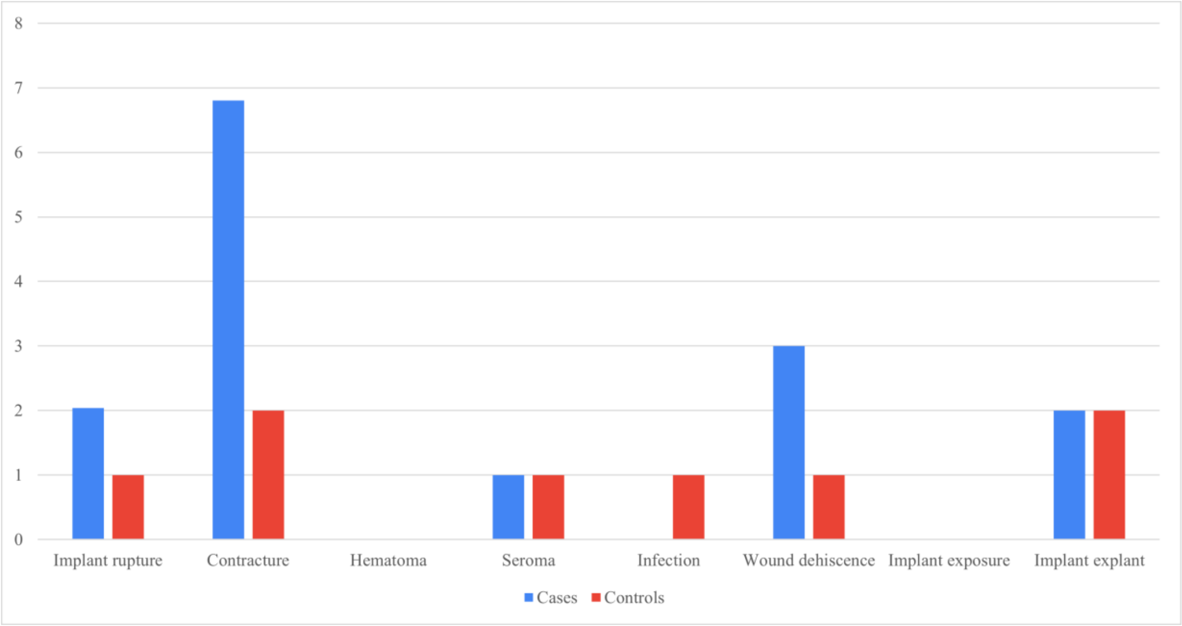

Figure S3. Late complications (Data presented as percentages. Y-axis scaled to data range for readability).

Table S1. Demographic analysis.

	Group 1 (Cases)	Group 2 (Controls)	P value
Total number	147	219	N.A.
Age (mean)	49	53	0,0004*
BMI (mean)	24,31	27,05	0,0536
Menopausal women	41% (60)	54% (118)	N.A.
Smoking habit			
Active smokers	22% (33)	12% (26)	N.A.
Stop smoking > 10 years	9% (13)	8% (18)	N.A.
Non smokers	69% (101)	80% (175)	N.A.
Diabetes	1% (2)	4% (9)	N.A.
Hypothyroidism	14% (21)	11% (25)	N.A.
Hypercholesterolemia	9% (13)	9% (19)	N.A.
Arterial hypertension	14% (21)	21% (47)	N.A.
*Legend: N.A. – not applicable; * - statistically significant at T-test*			

Table S2. Peri-operative hemogram analysis.

	**Group 1 (Cases)**	**Group 2 (Controls)**	**P value**
Pre-operative			
Hematocrit (mean)	39,21%	39,77%	0,0233*
Hemoglobin (mean)	13,31g/dL	13,43 g/dL	0,2191
Post-operative			
Hematocrit (mean)	34,49%	34,61%	0,3826
Hemoglobin (mean)	11,75 g/dL	11,56 g/dL	0,3955
Delta hemoglobin (mean)	1,57	1,66	0,4868
*Legend: * - statistically significant at T-test*			

Table S3. Oncological analysis.

	**Group 1 (Cases)**	**Group 2 (Controls)**
Tumor hystotype		
DCIS	15% (22)	11% (24)
IDC	51% (75)	62% (136)
LCIS	0% (0)	0,5% (1)
ILC	15% (22)	16% (34)
BRCA1	5% (7)	4% (8)
BRCA2	5% (8)	0,5% (1)
Staging		
T - Tumor		
pT0	2% (3)	1% (2)
pTis	13% (19)	7% (16)
1mi	5% (8)	4% (8)
1a	9% (13)	5% (12)
1b	8% (12)	10% (21)
1c	16% (24)	23% (51)
2	25% (36)	31% (67)
3	6% (9)	4% (9)
4a	0% (0)	0,5% (1)
4b	1% (1)	1% (3)
4c	0% (0)	0% (0)
4d	0% (0)	0,5% (1)
N - Nodes		
0	67% (98)	58% (128)
1	13% (19)	20% (44)
2a	1% (2)	4% (9)
2b	0% (0)	0% (0)
3a	4% (6)	4% (9)

3b	0% (0)	0,5% (1)
3c	0% (0)	0% (0)
M – Metastasis		
0	86% (126)	89% (196)
1	0% (0)	0% (0)
Receptor analysis		
ER+	71% (104)	68% (148)
PgR+	58% (85)	55% (120)
HER2+	12% (17)	20% (43)
Legend: DCIS – Ductal carcinoma in situ; LCIS – Lobular carcinoma in situ; IDC – Invasive ductal carcinoma; ILC – Invasive lobular carcinoma; BRCA – Breast Cancer mutation gene; ER – Estrogen Receptor; PgR – Progesteron Receptor; HER2 – Human Epidermal growth factor Receptor 2		
